# A chain mediation model reveals the association between family sense of coherence and quality of life in caregivers of advanced cancer patients

**DOI:** 10.1038/s41598-024-61344-4

**Published:** 2024-05-10

**Authors:** Panpan Cui, Chunyan Cheng, Huiying An, Xinyi Chen, Changying Chen, Hengyu Hu

**Affiliations:** 1 Department of Nursing, Henan Provincial Key Medicine Laboratory of Nursing, Henan Provincial People’s Hospital, Zhengzhou University People’s Hospital, No. 7 Weiwu Road, Zhengzhou, China; 2 School of Nursing, Zhengzhou University, Zhengzhou, China; 3 Hematology Department, Henan Provincial People’s Hospital, Zhengzhou University People’s Hospital, Zhengzhou, China; 4 Gastroenterology, Henan Provincial People’s Hospital, Zhengzhou University People’s Hospital, Zhengzhou, China; 5 Medical Oncology, Henan Provincial People’s Hospital, Zhengzhou University People’s Hospital, Zhengzhou, China; 6 The First Affiliated Hospital of Zhengzhou University, No. 1 Jianshe Dong Road, Zhengzhou, China; 7 Institute for Hospital Management of Henan Province, Zhengzhou, China

**Keywords:** Advanced cancer caregivers, Family sense of coherence, Psychological resilience, Psychological distress, Quality of life, Chain mediation, Quality of life, Cancer

## Abstract

Caregivers of advanced cancer patients face challenges impacting their quality of life (QoL). While evidence suggests that family sense of coherence (FSOC) can enhance individual psychological well-being and reduce distress symptoms, the precise mechanism through which FSOC improves caregivers' QoL remains unclear. This study aimed to explore the relationships among FSOC, psychological resilience, psychological distress, and QoL in primary caregivers of advanced cancer patients. A cross-sectional observational study was undertaken from June 2020 to March 2021 across five tertiary hospitals in China. Instruments included a general characteristic questionnaire, the Family Sense of Coherence Scale, the Patient Health Questionnaire-4, the 10-item Connor–Davidson Resilience Scale, and the 8-item SF-8 health survey. Pearson’s correlation and chain mediation analyses were performed using IBM SPSS (version 21) and PROCESS macro (version 3.4). Out of 290 valid questionnaires, results demonstrated that FSOC directly and positively influences caregivers' QoL. Psychological distress partially mediated the FSOC-QoL association, with paths "FSOC-psychological distress-QoL" and "FSOC-psychological resilience-psychological distress-QoL" contributing 43.08% and 6.72% of the total effect, respectively. Furthermore, this study distinguished physical and mental aspects of QoL, confirming both conform to the chain mediation model. FSOC impacts caregivers' QoL directly and indirectly through the mediation of psychological distress and the chain mediation effect of "psychological resilience-psychological distress". These insights enhance our understanding of the complex interplay between FSOC and QoL, underscoring the potential benefits of bolstering FSOC to strengthen caregiver resilience, alleviate distress, and ultimately elevate their QoL.

## Introduction

Cancer has become increasingly prevalent as a non-communicable disease. Its incidence has surged notably in recent times, marking it as a prominent global public health issue^1^. Caregivers, particularly those attending to advanced cancer patients, bear profound responsibilities^2^. Their roles span from providing medical care to offering emotional support and aiding in daily tasks^3^. Patients with advanced cancer, especially those in clinical stages III and IV, frequently present with multifaceted symptoms and a deteriorating health trajectory, often marked by resistance to curative treatments^4^. It's worth highlighting that the well-being of these patients often directly reflects their caregivers' quality of life (QoL)^5^. Numerous studies emphasize the diminished QoL experienced by caregivers of advanced cancer patients^6–8^, a decline that can potentially affect the quality of care provided, further impacting patient outcomes^9^. As such, understanding the determinants affecting caregivers' QoL is crucial, for their well-being is intrinsically linked to patient care quality.

### Family sense of coherence and quality of life

QoL encompasses physical and social functioning, along with perceived physical and mental well-being^10^. It reflects an individual’s satisfaction and sense of happiness with life, constituting a comprehensive concept influenced by multiple factors. Research indicates that caregivers of advanced cancer patients tend to have a heavier burden and diminished QoL compared to those caring for patients with other chronic conditions^11,12^. While earlier research primarily focused on individual-level factors influencing caregivers' QoL^13^, there has been a limited exploration of family-level determinants, such as family sense of coherence (FSOC). This concept, evolved from individual-level sense of coherence, embodies the family's collective comprehension of cancer-induced stress and their perceived ability to manage it^14^. It emphasizes the cohesion within the family and their collective ability to cope with stress. A robust FSOC might empower both the patient and caregiver to view cancer more as a challenge than a tribulation, maintaining familial stability amid the disruptions caused by the disease, and finding meaning in the journey, thus bolstering overall family resilience^15^.

Findings from Ngai et al. revealed FSOC’s direct positive impact on the QoL of infertile couples, mitigating stress-induced effects on both partners^16^. Such insights suggest that FSOC could be pivotal in enhancing individual QoL, a relationship yet to be thoroughly studied in the context of chronic illness and caregiving. A collective family approach to interpreting and navigating stressors, underpinned by a belief in deriving purpose from such challenges, could bolster caregiver mental well-being.

Mollerberg's study, which extended the FSOC framework to caregivers of advanced cancer patients in palliative care, identified a positive correlation between FSOC and hope, and a negative one with levels of anxiety and depression^17^. Though these insights underscore the interplay between FSOC, psychological well-being, and QoL, this nexus remains relatively uncharted in chronic disease settings, especially among advanced cancer cohorts. The intricate mechanisms by which FSOC influences QoL are yet to be fully clarified. Consequently, this research posits Hypothesis 1: FSOC exerts a direct and positive impact on the QoL of caregivers tending to advanced cancer patients.

### The mediating role of psychological resilience

The intricate mechanisms underlying how FSOC influences QoL warrant further investigation, with psychological resilience potentially acting as a mediator. Psychological resilience is characterized by an individual's adeptness in navigating adversity, trauma, or threats^18^. It emphasizes an individual's capability to adapt, rebound, and sustain mental equilibrium in the face of life's multifaceted challenges. Such resilience not only facilitates navigation through tough scenarios but also fosters the maintenance of a positive emotional state, enabling preservation or restoration of regular life functions. This makes psychological resilience a pivotal protective factor for QoL.

A recent systematic review elucidated that caregivers of advanced cancer patients initiate their psychological resilience process from the point of diagnosis, which could culminate in psychological well-being, benefit finding, and personal growth^19^. The coping strategies employed by caregivers throughout the caregiving trajectory represent a spectrum of means to modulate this resilience process. Within this spectrum, FSOC emerges as a significant family-level coping strategy. While the nexus between FSOC and individual psychological resilience has yet to be extensively probed, studies focused on psychological resilience in cancer patients have underscored a robust positive correlation between individual-level sense of coherence and resilience^20,21^. The dynamic between family-level sense of coherence and psychological resilience, however, remains ambiguous.

Theoretically, caregivers boasting higher degrees of FSOC likely benefit from fortified emotional bonds within the family^14^. Such bonds can imbue caregivers with amplified confidence and fortitude to tackle caregiving challenges. This cohesive family environment at the familial level might also bolster unity in patient care, fostering collective optimism, motivation, and hope^17^. In turn, this can boost caregivers' psychological resilience, augmenting their QoL. Consequently, we posits Research Hypothesis 2: FSOC exerts a direct influence on QoL, with psychological resilience serving as a partial mediator in this dynamic.

### The mediating role of psychological distress

Psychological distress presents as a form of psychological discomfort that may affect an individual’s emotions, behaviors, and daily functioning. It stands as a critical determinant of the QoL for caregivers of advanced cancer patients. Previous studies attest that such caregivers frequently grapple with elevated levels of psychological distress, encompassing symptoms like anxiety and depression^22,23^. These emotional strains can be attributed to the multifaceted responsibilities of caregiving, the patient's deteriorating health, and the uncertainties looming about the future^24^, often varying in tandem with the patient's physical well-being^25^.

Evidence reveals that manifestations of psychological distress can markedly undermine caregivers' QoL across several facets, including physical and mental health, social interactions, and holistic well-being^26^. The erosion in QoL due to these mental health challenges might stem from the onerous demands of caregiving, the emotional toll of witnessing patient suffering, feelings of despondency and impotence amidst dim recovery prospects for the patient, a paucity of social support systems for emotional unburdening, and the sidelining of self-care in favor of patient-centric concerns, among other dynamics^27^.

FSOC, as a family-level coping mechanism, encapsulates the collective cognizance of the stress induced by advanced cancer^14^, the conviction in harnessing familial resources to navigate this stress, and the discernment of purpose within these taxing circumstances. According to related theory, a heightened sense of coherence within the family typically propels members to adopt a collaborative stance towards stress mitigation, fostering mutual support and collaborative problem-solving^28,29^ This collaborative milieu equips caregivers to adeptly manage caregiving challenges. Concurrently, the shared emotional landscape facilitates positive emotional coping, thereby dampening psychological distress and bolstering caregivers' QoL. Consequent to the above elucidation, we advances Hypothesis 3: psychological distress acts as a mediator in the nexus between FSOC and QoL.

### Chain-mediation effects of psychological resilience and psychological distress

The family systems theory underscores the interconnectedness and interdependence inherent within families^30^. It posits that familial interactions and relationships play a pivotal role in determining the collective functionality and well-being of the unit. Within this theoretical framework, FSOC emerges as a crucial element. A heightened sense of cohesion fosters a family's resilience against external stressors, culminating in positive health outcomes for its members, caregivers included^15^. Such coherence equips caregivers with a nurturing, stable environment, facilitating their ability to navigate challenges and bolstering their psychological resilience.

The social support theory delineates the profound influence of social bonds on individual health outcomes, such as psychological distress^31^. This support can emanate from diverse sources, including family, friends, and broader social networks^32^. Through this lens, FSOC can be perceived as a specialized form of social support, dispensing emotional understanding and solace within familial confines. Given the strenuous nature of caregiving responsibilities, caregivers inevitably lean on social support structures to mitigate the associated stresses^33^. Under this paradigm, robust FSOC translates to enriched emotional sustenance and adaptive coping tools, aiding caregivers in countering psychological distress.

The psychological resilience theory views sense of coherence as a valuable resource^34^. When there's mutual support and comprehension within a family, it fosters an environment conducive for individuals to cultivate elevated psychological resilience. This heightened resilience, in turn, equips them to deftly handle caregiving duties and associated challenges, potentially alleviating their psychological distress. Existing literature posits that individual resilience plays a decisive role in mitigating psychological distress, notably anxiety and depression^35,36^, which is a risk factor for QoL. When a sense of coherence extends to the family level, referred to FSOC, it has the potential to improve QoL through psychological resilience and psychological distress.

**Figure 1:**
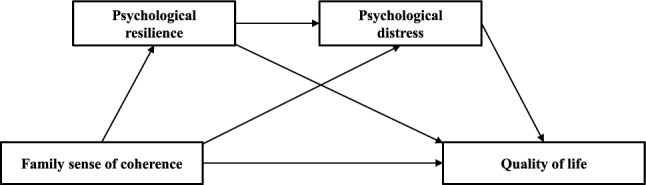
Hypothesized conceptual model of the chain mediation.

Aligned with the theories discussed above, it is anticipated that better FSOC may correlate with higher levels of psychological resilience among caregivers. This, in turn, is expected to be linked with lower levels of psychological distress, thereby potentially improving QoL. Understanding how FSOC influences QoL helps illuminate how caregivers of advanced cancer patients utilize family resources for psychosocial adjustment from a familial standpoint. This not only provides a more comprehensive perspective but also lays the groundwork for designing interventions that incorporate family factors, potentially enhancing their effectiveness. Nevertheless, the literature offers a limited exposition on the intertwined roles of psychological resilience and psychological distress as intermediaries in the relationship between FSOC and caregivers' QoL, especially concerning caregivers of advanced cancer patients—a demographic inherently susceptible and in dire need of robust support structures. Hence, synthesizing insights from the aforementioned theories and existing research hypotheses, this study introduces Hypothesis 4. It postulates that, for caregivers of advanced cancer patients, psychological resilience and psychological distress exhibit chain-mediation effects in the nexus between FSOC and QoL, as depicted in the mediation model hypothesis (refer to Fig. 1). Furthermore, this study expands the model to incorporate two critical aspects of QoL: the physical health domain and the mental health domain.

## Methods

### Study design and settings

Between June 2020 and March 2021, a cross-sectional investigation was carried out across five oncology units in tertiary hospitals located in Henan Province, China.

### Participants and procedure

For inclusion in the study, advanced cancer patient caregivers had to meet the following criteria: (1) They provided care for stage IV cancer patients aged 18 or above; (2) They themselves were at least 18 years old; (3) Patients identified them as their main, non-professional caregiver; (4) They willingly gave informed consent. However, those caregivers with severe mental or physical health issues were not considered. Targeting a participant group of over 200, in line with recommendations for structural equation modeling^37^, 330 caregivers were approached, and 290 agreed to be part of our study.

The recruitment process utilized convenience sampling executed by five skilled research assistants, all registered nurses, each affiliated with a distinct hospital. If participants struggled with the survey, these assistants helped by reading questions out and recording answers impartially. Patient-related clinical details were diligently obtained from the hospital's record system by these research assistants. To maximize participant focus and response quality, surveys were typically handed out during afternoons, when fewer treatments were scheduled. Moreover, assistants were on standby to elucidate any unclear aspects, guaranteeing participants' thorough understanding of survey queries. Upon survey conclusion, assistants carefully checked for incomplete sections, promptly asking for any missed answers. Surveys with over 10% unanswered sections or evident response patterns were disqualified from the study to maintain data quality. This study adhered to the Declaration of Helsinki, receiving ethical clearance from the ethics committee of Zhengzhou University (number: ZZUIRB 2021-19). Before completing the questionnaire, all caregivers gave their informed consent. The questionnaires were anonymous; initials of participants’ names were used for recording and coding.

## Measurements

### *Sociodemographic* characteristics

We collected comprehensive sociodemographic data from caregivers, encompassing age, gender, marital status, education background, employment status, place of residence, average monthly family income per capita, existence of any long-term illnesses, relationship to the patient, prior caregiving experience, caregiving arrangement (sole responsibility or shared), duration of caregiving, and daily caregiving hours. These were considered as potential influencing factors on caregivers' QoL and used as control variables in subsequent analyses.

### Quality of life

We utilized the 8-item SF-8 health survey to gauge the QoL of caregivers^38^. Questions like “How would you rate your health over the previous 4 weeks?” were presented. Each query employed a Likert scale of either 5 or 6 levels, corresponding to a distinct health aspect. Individual scores were then transformed into T-scores (average = 50, standard deviation = 10), with a spectrum between 0 and 100. The SF-8 survey provides two composite scores: the Physical Component Summary (PCS) and the Mental Component Summary (MCS)^39^. Higher average scores across all dimensions represent superior health. This survey yielded a Cronbach’s alpha of 0.871 in our research.

### Family sense of coherence

Using the Family Sense of Coherence Scale (FSOC-S) short form, we assessed the sense of coherence of the family in caregivers^40^. It comprises 12 items, scored on a 7-point Likert scale. Total scores can range from 12 to 84, with ascending scores reflecting enhanced family coherence. The Chinese version of FSOC-S showed a Cronbach’s α of 0.83, test–retest reliability of 0.75, and a CVI exceeding 0.9 for every item^41^. Cronbach’s α value in our cohort was 0.760.

### Psychological resilience

For evaluating psychological resilience among caregivers, we employed the 10-item Connor–Davidson Resilience Scale (CD-RISC-10), designed by Campbell-Sills^42^. This instrument uses a singular dimension with items scored between 0, representing "not true at all," and 4, indicating "true nearly all the time." Cumulative scores can span from 0 to 40, with higher scores denoting stronger resilience. For the Chinese adaptation used with cancer caregivers, the Cronbach’s α stood at 0.877, the test–retest reliability was 0.73, and the CVI for each item ranged from 0.83 to 1^43^. In our study cohort, the Cronbach’s α achieved a value of 0.906.

### Psychological distress

To measure psychological distress, we adopted the 4-item Patient Health Questionnaire (PHQ-4)^44^. Comprising two subsets focused on depression and anxiety, respondents clarified their feelings over the recent fortnight, with a scoring system from 0 (“unaffected”) to 3 (“consistently affected”). Cumulative scores for both subsets range from 0 to 6, with the entire PHQ-4's span being 0–12. The tool's reliability, as measured by the Cronbach’s α, was 0.862 in this investigation.

### Clinical patient data

Research assistants extracted pertinent clinical information about patients, such as age, gender, type of primary cancer, and the duration of advanced cancer, from the healthcare database.

### Statistical analysis

Descriptive analysis was performed to scrutinize the data. All demographic variables, including gender and education level, were categorized and summarized using frequencies and percentages. Continuous variables, such as scale scores, were expressed as means with standard deviations (mean ± SD). To investigate the interrelationships between different variables, *Pearson* correlation analysis was utilized. The impact of various factors on the QoL and its specific domains for caregivers was assessed using independent t-tests and one-way ANOVA where appropriate. Further analysis entailed a chain mediation using PROCESS 3.4 macro within SPSS 21.0, selecting Model 6. In this analysis, FSOC was the independent variable, with QoL— encompassing the physical and mental health domains— as the dependent variables, while psychological resilience and psychological distress served as mediators. Control variables included any statistically significant characteristics from the univariate analysis. The Bootstrap method, with a sample size of 5000, was employed to assess mediation effects^45^. A mediation effect is deemed significant if its confidence interval does not encompass 0. For the purposes of this study, a significance level of *P* < 0.05 was adopted, with α designated for a two-tailed test.

## Results

### Common method deviation test

Harman single-factor test was performed to check for common method bias^46^. The results showed that there were 7 factors with characteristic roots greater than 1, and the variance explained by the first factor was 29.13%, less than the critical standard of 40%. Therefore, there are no serious common methodological biases in this study and it can be continued.

### Descriptive statistics

Out of the 300 returned questionnaires (90.9%), 290 (representing 87.9%) were found to be valid for analysis. Table 1 shows the demographic characteristics of the subjects and a univariate analysis of QoL scores and their sub-dimensions across various characteristics. The mean age was 55.4 years for patients and 44.6 years for caregivers. Female caregivers made up 52.1% of the sample, and most were married. On the employment front, 43.8% of the caregivers had jobs. Nearly half, or 49.7%, indicated a monthly per capita household income exceeding 3000 RMB, and 50.7% hailed from rural settings. In the patient group, 76.2% had been diagnosed primarily with a solid tumor, and the median duration post-advanced cancer diagnosis was eight months. Approximately half of the caregivers had dedicated a minimum of 6 months to patient care. Statistically significant differences were observed in caregivers’ QoL and physical health scores relative to education level, working status, the presence of chronic diseases, monthly income, and prior caregiving experience (*P* < 0.05), while working status and monthly income showed significant differences in the mental health scores of caregivers (*P* < 0.05).

**Table 1:** Univariate analysis of quality of life and its sub-dimensions of caregivers with different characteristics (N = 290).

Variables	Group	N (%)	Quality of life		Physical health		Mental health	
			Mean ± SD	***F*/*t***	Mean ± SD	***F*/*t***	Mean ± SD	***F*/*t***
*Patient characteristics*								
Age (year)	< 45	64 (22.1)	80.51 ± 15.78	0.586	81.07 ± 14.97	1.929	79.58 ± 19.91	0.061
	45 ~ 59	102 (35.2)	80.54 ± 15.17		80.80 ± 14.77		80.11 ± 18.46	
	≥ 60	124 (42.7)	78.33 ± 15.27		76.94 ± 15.97		80.65 ± 17.77	
Sex	Male	159 (54.8)	78.98 ± 14.99	− 0.114	78.42 ± 15.67	− 0.378	79.93 ± 17.39	0.277
	Female	131 (45.2)	79.19 ± 15.77		79.11 ± 15.27		79.33 ± 19.57	
Primary cancer	Solid tumor	221 (76.2)	78.62 ± 14.92	− 0.900	78.13 ± 14.97	− 1.185	79.45 ± 18.35	− 0.341
	Hematologic tumor	69 (23.8)	80.53 ± 16.57		80.65 ± 16.95		80.31 ± 18.58	
Time since advanced cancer diagnosis (month)	< 8 months^┼^	137 (47.2)	80.51 ± 15.16	1.508	80.23 ± 14.60	1.573	80.96 ± 19.41	1.146
	≥ 8 months	153 (52.8)	77.79 ± 15.40		77.38 ± 16.13		78.49 ± 17.38	
*Caregiver characteristics*								
Age (year)	< 45	151 (52.1%)	80.54 ± 15.01	0.615	81.21 ± 15.14	1.880	79.41 ± 18.13	0.570
	45 ~ 59	99 (34.1%)	78.17 ± 17.30		77.06 ± 16.87		80.02 ± 20.58	
	≥ 60	40 (13.8)	80.25 ± 11.29		78.54 ± 11.61		83.11 ± 14.50	
Sex	Male	139 (47.9)	78.73 ± 15.44	− 0.370	78.52 ± 15.54	− 0.221	79.08 ± 18.96	− 0.514
	Female	151 (52.1)	79.40 ± 15.25		78.92 ± 15.45		80.19 ± 17.87	
Marital status	Married	255 (87.9)	79.01 ± 14.53	− 0.141	78.44 ± 14.79	− 0.677	79.97 ± 17.51	0.780
	Unmarried/divorced/widowed	35 (12.1)	79.52 ± 20.44		80.80 ± 19.87		77.38 ± 23.96	
Education Level	Junior high school and below	128 (44.1)	76.51 ± 16.60	3.379*	75.94 ± 16.43	4.242*	77.47 ± 19.82	1.640
	High school/Vocational school	84 (29.0)	80.53 ± 14.18		79.86 ± 15.14		81.65 ± 15.78	
	College and above	78 (26.9)	81.71 ± 13.77		82.09 ± 13.41		81.09 ± 18.35	
Working status	Employed	127 (43.8)	81.90 ± 13.87	2.406*	81.42 ± 14.36	2.270*	82.70 ± 16.41	2.158*
	Unemployed/retired	163 (56.2)	77.45 ± 15.90		77.17 ± 15.90		77.90 ± 19.24	
Place of residence	Rural	147 (50.7)	79.14 ± 16.76	0.069	78.80 ± 17.00	0.076	79.71 ± 19.16	0.047
	Urban	143 (49.3)	79.01 ± 13.74		78.66 ± 13.77		79.60 ± 17.60	
The presence of chronic diseases	Yes	52 (17.9)	72.30 ± 16.46	3.594**	70.48 ± 15.68	4.375**	75.32 ± 20.41	1.886
	No	238 (82.1)	80.56 ± 14.68		80.53 ± 14.85		80.60 ± 17.81	
Average monthly family income per capita (RMB)	< 3000	146 (50.3)	76.75 ± 16.44	− 2.628**	76.57 ± 16.48	− 2.413*	77.05 ± 19.37	− 2.447*
	≥ 3000	144 (49.7)	81.43 ± 13.76		80.92 ± 14.08		82.29 ± 16.98	
Relationship to the patient	Spouse	121 (41.7)	80.48 ± 13.75	0.746	79.54 ± 13.86	0.754	80.03 ± 18.27	0.292
	Non-spouse	169 (58.3)	79.00 ± 16.57		78.15 ± 16.54		79.39 ± 18.50	
Prior caregiving experience	Yes	68 (23.4)	75.86 ± 16.26	1.986*	75.35 ± 16.48	2.068*	76.72 ± 18.80	1.511
	No	222 (76.6)	80.06 ± 14.92		79.76 ± 15.03		80.56 ± 18.19	
Caregiving arrangement	Care for patients alone	159 (54.8)	80.15 ± 16.55	1.322	80.29 ± 16.14	1.903	79.93 ± 20.03	0.277
	Care for patients with others	131 (45.2)	77.77 ± 13.62		76.83 ± 14.44		79.33 ± 16.22	
Duration of caregiving (month)	< 6	158 (54.4)	80.13 ± 14.47	1.702	79.94 ± 14.38	1.835	80.43 ± 17.74	1.059
	6 ~	66 (22.8)	79.58 ± 16.47		78.92 ± 16.95		80.68 ± 19.29	
	≥ 12	66 (22.8)	76.05 ± 15.96		75.62 ± 16.23		76.77 ± 18.92	
Daily caregiving hours (h)	< 6	96 (33.1)	79.28 ± 14.96	0.152	78.36 ± 15.01	0.146	80.82 ± 18.10	0.341
	7 ~	79 (27.2)	78.35 ± 14.20		78.22 ± 14.80		78.59 ± 16.92	
	12 ~	37 (12.8)	78.43 ± 18.99		78.73 ± 18.76		77.93 ± 22.50	
	18–24	78 (26.9)	79.86 ± 15.19		79.69 ± 15.23		80.13 ± 18.23	

*SD* standard deviation. For dichotomous variables, independent samples *t* tests were used while one-way ANOVA was used for variables with three or more categories. *P < 0.05; **P < 0.01.

### Correlation analysis of FSOC, psychological resilience, psychological distress, and QoL

Table 2 presents the mean values, SDs, and correlations among the variables. The QoL scores averaged 79.08 with a SD of 15.32. FSOC demonstrated a positive correlation with both psychological resilience (*r* = 0.350, *P* < 0.01) and QoL and its sub-dimensions (*r* = 0.318–0.389, *P* < 0.01), and a negative correlation with psychological distress (*r* = − 0.332, *P* < 0.01). Additionally, psychological resilience correlated positively with QoL and its sub-dimensions (*r* = 0.203–0.244, *P* < 0.01) and negatively with psychological distress (*r* = − 0.253, *P* < 0.01). Notably, a strong negative correlation was observed between psychological distress and QoL and its sub-dimensions (*r* = − 0.641 to − 0.499, *P* < 0.01). These significant correlations among variables support the investigation of subsequent hypotheses. To address concerns about the high correlation between psychological distress and the mental health dimension in QoL possibly being due to correlated items in the two scales, additional correlation analyses were conducted between the four items of psychological distress and two dimensions of QoL. The results indicated that all four items of the PHQ-4 scale exhibited a significant negative correlation with both the mental health and physical health dimensions of QoL, not solely due to the high correlation between psychological distress and the mental health dimension.

**Table 2:** Correlations among family sense of coherence, psychological resilience, psychological distress, and quality of life.

Variables	QoL	PH	MH	FSOC	PR	PD
QoL	1.00					
PH	0.948**	1.00				
MH	0.894**	0.704**	1.00			
FSOC	0.389**	0.389**	0.318**	1.00		
PR	0.244**	0.242**	0.203**	0.350**	1.00	
PD	− 0.603**	− 0.499**	− 0.641**	− 0.332**	− 0.253**	1.00
Mean	79.08	78.73	79.66	59.50	30.56	2.91
SD	15.32	15.47	18.38	11.24	8.95	2.63

*FSOC* family sense of coherence, *MH* mental health, *PD*: psychological distress, *PH* physical health, *PR* psychological resilience, *QoL* quality of life, *SD* standard deviation. **P < 0.01.

### Test of mediation

**Figure 2:**
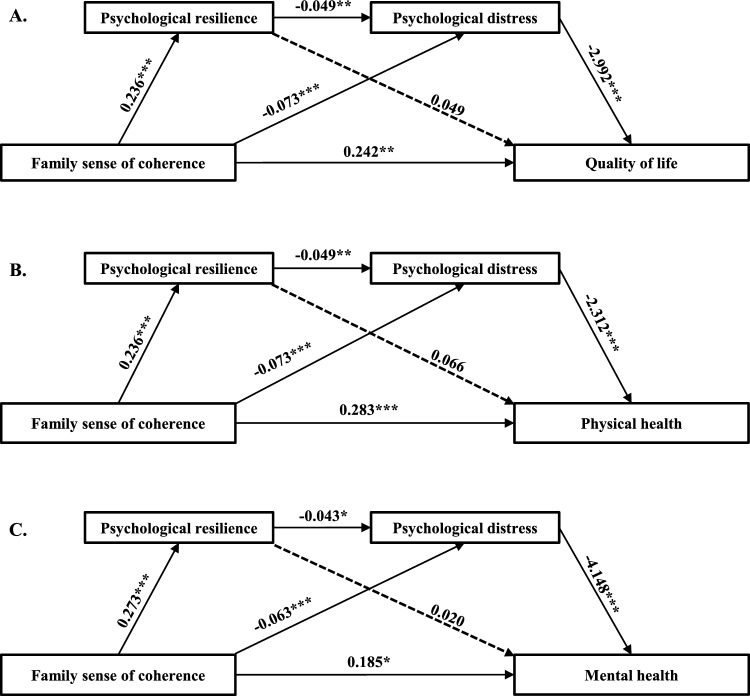
The mediation models. Model (**A**) and (**B**) were adjusted by caregivers’ education level, working status, the presence of chronic diseases, average monthly family income per capita, and prior caregiving experience. Model (**C**) was adjusted by caregivers’ working status and average monthly family income per capita. * *P* < 0.05; ** *P* < 0.01; *** *P* < 0.001.

The results generated by PROCESS macro are presented in Tables 3, 4 and Fig. 2. Mediation analyses investigated the relationships between FSOC and QoL, as well as between FSOC and physical health, controlling for education level, working status, the presence of chronic diseases, monthly income, and prior caregiving experience. FSOC emerged as a positive predictor of psychological resilience (*β* = 0.236, *P* < 0.01) and a negative predictor of psychological distress (*β* = − 0.073, *P* < 0.001), as indicated in Table 3. The direct effects of FSOC on QoL and physical health were both statistically significant (*β* = 0.242, *P* < 0.01; *β* = 0.283, *P* < 0.001, respectively). Additionally, psychological resilience negatively influenced psychological distress (*β* = − 0.049, *P* < 0.01) and psychological distress was strongly inversely associated with both QoL and physical health (*β* = − 2.992, *P* < 0.001; *β* = − 2.312, *P* < 0.001, respectively). No direct correlations were observed between psychological resilience and QoL (*β* = 0.049, *P* > 0.05), nor between psychological resilience and physical health (*β* = 0.066, *P* > 0.05). After adjusting for working status and monthly income, FSOC also positively predicted psychological resilience (*β* = 0.273, *P* < 0.001) and negatively predicted psychological distress (*β* = − 0.063, *P* < 0.001) in relation to mental health, with FSOC’s direct effect on mental health being statistically significant (*β* = 0.185, *P* < 0.05). Psychological resilience again demonstrated a negative impact on psychological distress (*β* = − 0.043, *P* < 0.05), and psychological distress exhibited a strong negative correlation with mental health (*β* = − 4.148, *P* < 0.001), as depicted in Table 4.

**Table 3:** Testing the mediation effect of FSOC on QoL and physical health.

Outcome variable	Variables	R^2^	F	β	Std. β	SE	t
Psychological resilience	Working status	0.152	8.455	0.869	0.047	1.107	0.785
	Average monthly family income per capita			0.422	0.024	1.103	0.382
	The presence of chronic diseases			− 1.956	− 0.084	1.303	− 1.503
	Education level			1.683	0.155	0.693	2.431*
	Prior caregiving experience			0.909	0.043	1.179	0.771
	FSOC			0.236	0.296	0.047	5.003***
Psychological distress	Working status	0.168	8.119	0.249	0.046	0.323	0.771
	Average monthly family income per capita			− 0.752	− 0.143	0.322	− 2.340*
	The presence of chronic diseases			0.324	0.047	0.381	0.851
	Education level			0.556	0.175	0.204	2.729**
	Prior caregiving experience			0.07	0.011	0.344	0.204
	FSOC			− 0.073	− 0.312	0.014	− 5.093***
	Psychological resilience			− 0.049	− 0.166	0.017	− 2.820**
Quality of life/Physical health	Working status	0.434^a^/0.354^b^	26.952^a^/19.230^b^	− 2.277^a^/− 1.926^b^	− 0.072^a^/− 0.060^b^	1.556^a^/1.679^b^	− 1.463^a^/−1.147^b^
	Average monthly family income per capita			0.384^a^/0.182^b^	0.013^a^/0.006^b^	1.564^a^/1.687^b^	0.246^a^/0.108^b^
	The presence of chronic diseases			− 4.934^a^/−7.160^b^	− 0.124^a^/− 0.178^b^	1.838^a^/1.983^b^	− 2.684**^a^/− 3.610***^b^
	Education level			0.572^a^/0.961^b^	0.031^a^/0.051^b^	0.995^a^/1.073^b^	0.575^a^/0.896^b^
	Prior caregiving experience			− 2.055^a^/− 2.068^b^	− 0.057^a^/− 0.057^b^	1.657^a^/1.787^b^	− 1.240^a^/− 1.157^b^
	FSOC			0.242^a^/0.283^b^	0.177^a^/0.205^b^	0.072^a^/0.078^b^	3.351**^a^/3.631***^b^
	Psychological resilience			0.049^a^/0.066^b^	0.029^a^/0.038^b^	0.085^a^/0.091^b^	0.584^a^/0.721^b^
	Psychological distress			− 2.992^a^/− 2.312^b^	− 0.513^a^/− 0.393^b^	0.287^a^/0.310^b^	− 10.432***^a^/− 7.472***^b^

*FSOC* family sense of coherence; a: Quality of life; b: Physical health; SE: Standard error; Std. β = Standard β. *P < 0.05; **P < 0.01; ***P < 0.001.

**Table 4:** Testing the mediation effect of FSOC on mental health.

Outcome variable	Variables	R^2^	F	β	Std. β	SE	t
Psychological resilience	Working status	0.128	13.967	0.177	0.010	1.087	0.163
	Average monthly family income per capita			1.325	0.074	1.053	1.258
	FSOC			0.273	0.342	0.044	6.161***
Psychological distress	Working status	0.142	11.79	0.147	0.027	0.317	0.462
	Average monthly family income per capita			− 0.477	− 0.091	0.308	− 1.548
	FSOC			− 0.063	− 0.269	0.014	− 4.584***
	Psychological resilience			− 0.043	− 0.148	0.017	− 2.513*
Mental health	Working status	0.430	42.815	− 2.830	− 0.074	1.812	− 1.561
	Average monthly family income per capita			0.641	0.018	1.767	0.363
	FSOC			0.185	0.113	0.081	2.271*
	Psychological resilience			0.020	0.010	0.100	0.205
	Psychological distress			− 4.148	− 0.593	0.338	− 12.259***

*FSOC* family sense of coherence; SE: Standard error; Std. β = Standard β. *P < 0.05; ***P < 0.001.

The mediation analysis elucidated that psychological distress partially mediates the relationship between FSOC and QoL, where FSOC impacts QoL via two pathways: (a) FSOC → Psychological distress → QoL, (b) FSOC → Psychological resilience → Psychological distress → QoL, with mediating effects calculated at 0.218 [(− 0.073) × (− 2.992)] and 0.034 [0.236 × (− 0.049) × (− 2.992)], respectively. Similarly, psychological distress also partially mediates the relationship between FSOC and both physical and mental health. For physical health, the two pathways identified were (a) FSOC → Psychological distress → Physical health, (b) FSOC → Psychological resilience → Psychological distress → Physical health, with mediating effects of 0.169 [(− 0.073) × (− 2.312)] and 0.027 [0.236 × (− 0.049) × (− 2.312)], respectively. For mental health, the pathways were (a) FSOC → Psychological distress → Mental health, (b) FSOC → Psychological resilience → Psychological distress → Mental health, yielding mediating effects of 0.261 [(− 0.063) × (− 4.148)] and 0.049 [0.273 × (− 0.043) × (− 4.148)], respectively. These pathways are visually represented in Fig. 2.

Decomposition of effects in Table 5 reveals that the direct effect (0.242) and the total indirect effect (0.264) accounted for 47.83% and 52.17% of the total effect (0.506), respectively. Within the total indirect effect (0.264), the mediation effect of psychological distress and the chain mediation effect of psychological resilience and psychological distress accounted for 43.08% and 6.72% of the total effect, respectively. Table 6 presents the decomposition of effects for FSOC on the sub-dimensions of QoL. In the domain of physical health, the total effect of FSOC was 0.494, with the total indirect effect comprising 42.71% of this effect; specifically, the mediation effect of psychological distress accounted for 34.21%, and the chain mediation effect of “psychological resilience → psychological distress” accounted for 5.46%. Regarding the mental health domain, the total effect of FSOC was 0.501, with the total indirect effect constituted 63.07% of this effect. Within this, the mediation effect of psychological distress contributed 52.09%, while the chain mediation effect of “psychological resilience → psychological distress” accounted for 9.78%.

**Table 5:** Effect decomposition of family sense of coherence on quality of life.

Model pathways	Effect value	Total effect ratio	Boot 95% CI	Significance
*Direct effect*				
FSOC → QoL	0.242	47.83%	(0.100, 0.384)	√
*Indirect effect*				
FSOC → PR → QoL	0.012	2.37%	(-0.027, 0.054)	–
FSOC → PD → QoL	0.218	43.08%	(0.120, 0.338)	√
FSOC → PR → PD → QoL	0.034	6.72%	(0.010, 0.063)	√
Total indirect effect	0.264	52.17%	(0.160, 0.377)	√
Total effect	0.506	100.00%	(0.352, 0.660)	√

*CI* confidence interval, *FSOC* family sense of coherence, *PD* psychological distress, *PR* psychological resilience, *QoL* quality of life. The model was adjusted for caregivers' education level, working status, average monthly family income per capita, the presence of chronic diseases and the previous caregiving experience.

**Table 6:** Testing the mediating effects of the two dimensions of quality of life (physical health, mental health).

Model pathways	Effect value	Total effect ratio	Boot 95% CI	Significance
*Direct effect*				
FSOC → Physical health	0.283	57.29%	(0.129, 0.436)	√
FSOC → Mental health	0.185	36.93%	(0.025, 0.345)	√
*Indirect effect*				
FSOC → PR → Physical health	0.015	3.04%	(-0.030, 0.062)	−
FSOC → PD → Physical health	0.169	34.21%	(0.084, 0.274)	√
FSOC → PR → PD → Physical health	0.027	5.46%	(0.007, 0.050)	√
FSOC → PR → Mental health	0.006	1.20%	(-0.048, 0.062)	−
FSOC → PD → Mental health	0.261	52.09%	(0.134, 0.399)	√
FSOC → PR → PD → Mental health	0.049	9.78%	(0.011, 0.092)	√
*Total indirect effect*				
FSOC → Physical health	0.211	42.71%	(0.121, 0.317)	√
FSOC → Mental health	0.316	63.07%	(0.191, 0.448)	√
Total effect				
FSOC → Physical health	0.494	100.00%	(0.339, 0.648)	√
FSOC → Mental health	0.501	100.00%	(0.321, 0.680)	√

*CI* confidence interval, *FSOC* family sense of coherence, *PD* psychological distress, *PR* psychological resilience. The model for physical health was adjusted for caregivers' education level, working status, average monthly family income per capita, the presence of chronic diseases and the previous caregiving experience, while the model for mental health was adjusted for caregivers' working status and average monthly family income per capita.

## Discussion

This research presents a chain mediation model to elucidate the underlying mechanisms connecting FSOC and QoL among caregivers of advanced cancer patients. Our findings reveal that psychological distress partially mediates the FSOC-QoL relationship. Additionally, a combined chain mediation effect is observed with both psychological resilience and distress influencing the association between FSOC and QoL. The model is further validated by the inclusion of QoL’s two facets—physical and mental health—both of which conform to the chain mediation model.

### The direct effect of FSOC on QoL

This study reveals that, even after controlling for variables, FSOC continues to have a significant direct positive effect on the QoL among caregivers of advanced cancer patients. Elevated FSOC levels correlate with higher QoL scores, indicating an improved QoL for caregivers. In contrast, a previous study by Möllerberg^17^ recorded an FSOC score of 68.3 ± 10.8 for caregivers of patients in the palliative phase of cancer. Our study yielded a score of 59.50 ± 11.24, which is lower. This discrepancy might stem from the inclusion of patients in earlier cancer stages in Möllerberg's research, whereas our study specifically targeted stage IV cancer patients. This specialization may result in elevated stress levels for families, manifesting in diminished FSOC scores.

Existing research underscores the impact of FSOC on the QoL of infertility-afflicted couples^47^, particularly concerning their mental health. Furthermore, FSOC has been pinpointed as a mediator in the relationship between stress and QoL^16^. Another study by Nagi^48^ emphasized the role of FSOC in fostering a sense of meaning among other populations, motivating individuals to leverage both intrinsic and extrinsic resources to address challenges stemming from stressful events. These insights confirm FSOC's pivotal role in managing stress, preserving functionality, bolstering subjective well-being, and enhancing overall QoL, with a special focus on mental health. Our findings extend this understanding by demonstrating that FSOC positively influences not just mental health, but also physical well-being, especially in caregiving scenarios within chronic disease contexts. This underlines the indispensable nature of familial support in boosting caregivers' QoL. Hence, clinical nursing professionals ought to champion stronger interconnectivity among caregivers and their family members, promoting the formation of supportive networks, fortifying FSOC, and proactively providing resources to uplift caregiver well-being.

### The mediating role of psychological resilience and psychological distress between FSOC and QoL

In addition to the observed direct effects, this study identified a partial mediating role of psychological distress between FSOC and QoL. Caregivers of advanced cancer patients, faced with intense caregiving duties and the challenges of a deteriorating patient condition and future uncertainties, often manifest elevated psychological distress levels^22,23^. Established research underscores the pronounced negative correlation between caregivers' psychological distress and their QoL^49^. FSOC has been documented to bolster an individual's hopefulness and exhibits a substantial negative association with anxiety and depressive symptoms^17^. Grounded in social support theory, familial support invariably results in enhanced individual health outcomes, inclusive of psychological well-being^50^. Within this context, FSOC emerges as an essential familial coping mechanism, correlating with diminished psychological distress among caregivers perceiving heightened FSOC. This is congruent with prior studies exploring the nexus between individual sense of coherence and distress^51^ as well as FSOC's relationship with depression^52^.

Drawing on theory and previous research, FSOC may improve caregivers' psychological distress in several ways: (1) by providing emotional support and understanding, thereby alleviating the stress of coping with caregiving tasks; (2) by offering methods to cope with and manage stressors related to advanced cancer, helping caregivers better handle their tasks and challenges. This aligns with other research findings regarding the negative relationship between an individual's sense of coherence and caregiving burden^53–55^. Moreover, (3) FSOC can guide caregivers to view the stressors of advanced cancer from a positive perspective, find meaning in them, and set goals. This, in turn, inspires caregivers to proactively mobilize internal and external resources to cope with challenges, a finding consistent with research in infertility couples^48^. In essence, this study posits that enhanced FSOC could lessen the experience of psychological distress, and caregivers with diminished psychological distress are more likely to report improved QoL, characterized by greater physical and mental well-being. In clinical practice, emphasizing and strengthening FSOC among caregivers can be seen as a crucial strategy to alleviate their psychological distress and enhance their QoL. By fostering a more harmonious, supportive, and understanding family environment, caregivers can experience improved psychological well-being, diminished caregiving strain, and a more effective fulfillment of caregiving duties.

Our research indicates that FSOC can serve as a positive predictor for caregivers' psychological resilience. This finding aligns with prior studies highlighting a positive correlation between an individual's sense of coherence and resilience^20,21^. FSOC embodies the positive interactions, mutual support, and understanding prevalent among family members. Such familial cohesion bolsters caregivers' confidence, enabling them to effectively navigate the complexities and challenges posed by advanced cancer^56^. Furthermore, FSOC correlates with positive emotional experiences, enhancing caregivers' psychological resilience. Contrarily, our findings suggest that psychological resilience does not exhibit a statistically significant predictive effect on QoL, diverging from previous studies^57^. The study also determined no notable mediating influence of psychological resilience on the relationship between FSOC and QoL. Potential reasons for these discrepancies could be the utilization of different measurement tools in our study or variations in the cancer severity among patients under the care of these caregivers. Notably, earlier study overlooked factors such as cancer stage, progression, and patient-specific characteristics^57^. To fully understand these disparities, further investigation is warranted.

FSOC may indirectly affect QoL by the chain mediating effects of psychological resilience and psychological distress. Our analysis demonstrates that FSOC significantly forecasts enhanced psychological resilience, which in turn inversely correlates with anxiety and depression. Consequently, this promotes better physical and mental health outcomes. This aligns with previous research findings, indicating that, through stress management interventions by bolstering resilience, the psychological distress and QoL of adolescent and young adult cancer survivors can be significantly improved, with these positive effects lasting at least 2 years^35^. It is posited that caregiver optimism and hopefulness increase when there is perceived family unity, thereby enhancing their psychological resilience and their ability to cope with the challenges of advanced cancer care^58^. This enhancement may manifest in proactive coping, a positive outlook, and improved problem-solving abilities^59^, which collectively diminish psychological distress and, thus, elevate QoL.

Moreover, a robust FSOC offers essential psychological backing to caregivers, fostering a profound sense of support and understanding. Our research indicates that the influence of FSOC on QoL via the "psychological resilience → psychological distress" pathway is less substantial than the direct effects of psychological distress. According to stress appraisal theory, individuals assess potential threats and their resources in response to stress^60^. FSOC, reflecting family support, provides emotional relief to caregivers, directly diminishing their psychological distress as they sense increased family unity. Conversely, while psychological resilience—an innate capacity to rebound from adversity^18^—is beneficial for managing stress, its direct effect on QoL may not be as immediate as that of FSOC. Our findings also suggest that enhancing FSOC could be more crucial than boosting inner resources like psychological resilience for directly alleviating negative psychological responses. Nevertheless, the significance of psychological resilience as a mediator in this dynamic is reaffirmed, suggesting that clinical practitioners should focus on bolstering both FSOC and psychological resilience to improve the psychological status and QoL of caregivers.

## Strengths and limitations

This study explores the impact of a family-level coping resource, namely FSOC, on the QoL of caregivers. Extending the existing body of research that has examined the relationship between an individual’s sense of coherence and health outcomes, our findings offer valuable insights for clinical practitioners seeking to enhance the support provided to caregivers of advanced cancer patients. Furthermore, we delve into the underlying mechanisms at play within the two dimensions of QoL (physical health and mental health). Our results confirm the applicability of a chain mediation model to these two dimensions. Therefore, enhancing family coherence can bolster individual resilience, reduce distress, and promote both physical and mental well-being. This holds significant promise for positively contributing to the overall health and well-being of caregivers within the context of advanced cancer.

However, several limitations exist. Firstly, the cross-sectional design constrains our ability to confirm causal links between variables and assess the long-terms effects of FSOC on caregiver outcomes. To address this, future studies should consider adopting longitudinal or experimental methodologies to deepen the understanding of these relationships. Additionally, conducting qualitative interviews with caregivers who have different relationships with advanced cancer patients could yield richer insights into the nuances of the caregiving experience and enhance the study’s findings. Although the study controlled for certain demographic variables, it’s important to acknowledge the potential influence of other confounding factors, including social support, coping strategies, cultural factors, and patient characteristics such as treatment status. Future research could benefit from a more comprehensive examination of these potential confounders. Moreover, while our research focused on the mediating roles of psychological resilience and distress, it's pivotal to recognize that the influence of FSOC on QoL might be shaped by other mediators or moderators, such as dyadic coping or family functioning, warranting further investigation. Furthermore, this study relied solely on self-report measures. Future studies could enhance the robustness of findings by employing a combination of self-report measures and objective assessments, such as physiological indicators and family environment assessments. Given that our samples primarily originated from tertiary oncology hospitals in one province through convenience sampling, there is a risk of selection bias, limiting the generalizability of findings to other healthcare settings or geographical locations. Future studies would benefit from a more diverse and representative sample base to facilitate holistic comparisons and long-term monitoring. Finally, assessing the applicability of the established mediation model to other chronic patient groups is an essential direction for future inquiries.

## Conclusion

In summary, this research elucidates the processes through which FSOC bolsters the QoL of caregivers attending to advanced cancer patients. Importantly, FSOC not only directly amplifies caregivers' QoL but also has an indirect influence mediated by psychological distress. Additionally, the study unveils a chain mediation effect encompassing both psychological resilience and distress, further emphasizing the multifaceted ways in which FSOC can enhance caregivers' well-being. These findings deepen our comprehension of the intricate relationship between FSOC and QoL, emphasizing the potential benefits of interventions targeting FSOC enhancement and distress reduction to promote caregiver QoL.

## Data Availability

Data and analytical methods in this study are available from the corresponding author upon reasonable request.
